# Machine Learning‐Assisted KCl‐CaCl_2_‐LiCl Electrolyte Design for Low‐Temperature, High‐Performance Calcium‐Based Liquid Metal Batteries

**DOI:** 10.1002/advs.75994

**Published:** 2026-06-03

**Authors:** Xinglin Zhou, Lei Huang, Yan Zhou, Xiaohui Ning

**Affiliations:** ^1^ Center For Alloy Innovation and Design (CAID) State Key Laboratory For Mechanical Behavior of Materials Xi'an Jiaotong University Xi'an Shaanxi P. R. China

**Keywords:** electrolyte design, liquid metal batteries, machine learning

## Abstract

Calcium‐based liquid metal batteries are promising for large‐scale energy storage due to calcium abundance and low cost, yet their practical applications are impeded by high operating temperatures, severe self‐discharge, limited coulombic efficiency, and rapid capacity fading. Here, we develop a machine learning (ML)‐assisted optimization framework, integrating data‐driven analysis, ML prediction, and experimental verification to design a high‐performance ternary molten‐salt electrolyte. Through multi‐parameter evaluation of thermodynamic stability, melting behavior, density, and cost, KCl was identified as an optimal third component for CaCl_2_‐LiCl based systems. A multidimensional descriptor‐performance dataset was constructed to develop a random forest model for precise electrolyte composition optimization. Guided by this model, the KCl‐CaCl_2_‐LiCl electrolyte (13:35:52 mol%) was experimentally verified to enable stable operation at 480°C, delivering a coulombic efficiency >99.5%, an ultralow self‐discharge current density of 0.31 mA cm^−2^, >91% capacity retention after 100 cycles, while maintaining a low material cost of 0.81$ kg^−1^. This optimized ternary electrolyte suppresses calcium dissolution through cooperative multi‐cation effects, significantly improving low‐temperature electrochemical performance and cycling stability. This work not only provides a viable pathway toward practical Ca‐based LMBs but also establishes a generalizable ML‐assisted paradigm for accelerated electrolyte design in advanced electrochemical energy storage.

## Graphical abstract

A machine learning‐assisted framework optimizes the KCl‐CaCl_2_‐LiCl ternary electrolyte. The optimized 13:35:52 mol% composition enables Ca‐based liquid metal batteries to operate stably at 480 °C, with >99.5% coulombic efficiency, ultralow self‐discharge, and excellent cycling stability, advancing low‐temperature large‐scale energy storage.

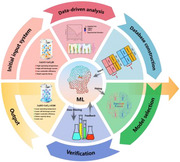

## 1 Introduction

The transition to a carbon‐neutral energy infrastructure hinges on safe, low‐cost, and instantly dispatchable energy storage technologies capable of stabilizing increasingly volatile renewable power systems. Large‐scale energy storage thus plays a pivotal role in buffering intermittent renewable electricity, enhancing grid resilience, and enabling high‐penetration deployment of clean energy resources [1, 2].

Among various candidate technologies, liquid metal batteries (LMBs) have garnered widespread attention owing to their intrinsically low cost, high safety, rapid reaction kinetics, and exceptionally long cycle life [3, 4]. Their fully liquid architecture comprising a liquid‐metal anode, a molten‐salt electrolyte, and a liquid‐metal cathodenaturally forms stable density‐stratified layers and sharp liquid‐liquid interfaces [5]. This fully liquid configuration enables fast ion transport, eliminates mechanical degradation associated with solid electrodes, and avoids dendrite formation, thereby ensuring robust performance under high‐current and long‐duration operation [6, 7, 8, 9, 10, 11].

Considering resource sustainability and environmental impact, calcium stands out as an ideal anode material. As the fifth most abundant element in the Earth's crust, it possesses multiple advantages such as low cost, non‐toxicity, low density (approximately 1.54 g cm^−3^), and a relatively negative standard reduction potential (‐2.87 V vs. NHE) [12]. Consequently, a variety of Ca‐based LMB electrode systems, including Ca||Sb [13, 14], Ca||Bi [15], Ca||Pb [16], CaMg||Bi [17] and CaLiMg||Bi [18] have been developed. However, pure calcium metal has a relatively high melting point (842°C) and exhibits large solubility in conventional halide molten‐salt electrolytes, imparting electronic conductivity in the electrolyte. These lead to severe self‐discharge, low coulombic efficiency, and accelerated active material loss, thereby preventing the practical deployment of Ca‐based LMBs [17].

To mitigate these issues, previous efforts mainly fell into two categories: first, optimizing cathode materials to adjust alloying thermodynamics [13, 14, 15, 16], and second, alloying calcium with additional metals to reduce operating temperatures and suppressing self‐discharging [17, 18]. For example, Ouchi et al. demonstrated that forming Ca‐Mg alloys significantly reduced self‐discharge current density from 10 to 4 mA cm^−2^ while preserving the original discharge voltage plateau [17].

However, electrode optimization alone remains insufficient. In high temperature molten‐salt systems, the electrolyte fundamentally governs Ca activity, dissolution behavior, ion transport, and electrochemical stability. Thus, developing new electrolytes with good chemical compatibility, low melting point, suppressed Ca dissolution, and low cost is imperative for enabling practical Ca‐based LMBs.

Pioneering studies explored CaF_2_ [13], followed by complex chloride mixtures such as LiCl‐NaCl‐CaCl_2_ and LiCl‐NaCl‐CaCl_2_‐BaCl_2_, which achieved improved efficiency at ∼600°C [15, 19]. After that, Ouchi et al. showed that introducing additional cations effectively reduces Ca^2+^ activity in the melt. The LiCl‐CaCl_2_ (65:35 mol%) electrolyte, which is widely adopted today, combined with Ca‐Mg anodes enabled operation at 550°C with reduced self‐discharge [17]. Nevertheless, the melting point of this binary electrolyte remains high, limiting further reduction of the operating temperature. High operating temperatures exacerbate calcium dissolution, leading to low coulombic efficiency and elevated self‐discharge current. Therefore, the rational design of molten‐salt electrolytes, capable of lowering operating temperature while simultaneously suppressing calcium solubility and maintaining high ionic conductivity, is critically important for advancing Ca‐based LMB performance.

Traditional trial‐and‐error approaches struggle in this multi‐variable, non‐linear compositional space. A more systematic and predictive strategy is required. Electrolytes in multi‐component molten‐salt systems exhibit complex thermodynamic and transport behaviors that are difficult to optimize by intuition alone. Machine learning (ML) provides an attractive pathway for exploring vast composition spaces and uncovering structure‐property relationships. To address these challenges, we combine multi‐parameter evaluation with ML‐assisted composition optimization to design a high‐performance ternary chloride electrolyte. By evaluating stability, melting point, density, and cost, we selected KCl as an effective third component for modifying the CaCl_2_‐LiCl binary melt. Based on an experimentally curated dataset, a random forest (RF) model was established to quantitatively predict the electrochemical performance of multi‐component electrolytes with pre‐defined compositions and guide high‐throughput composition refinement via progressive screening. Guided by this research framework, we successfully designed a KCl‐CaCl_2_‐LiCl electrolyte system that enables Ca‐based LMBs to operate at 480°C. The optimized composition provides a coulombic efficiency over 99.5%, a self‐discharge current density of only 0.31 mA cm^−2^, and 91% capacity retention over 100 cycles, while maintaining a material cost of 0.81$ kg^−1^. These improvements originate from multi‐cation synergistic effects that modulate calcium activity in the melt, reduce its solubility, and stabilize electrode‐electrolyte interfaces. This study not only provides a viable route toward the practical deployment of Ca‐based LMBs but also establishes a generalizable ML‐assisted paradigm for accelerated electrolyte design in advanced energy storage systems.

## 2 Results and Discussion

### 2.1 Design

To address the core performance bottlenecks of the Ca|LiCl‐CaCl_2_|Bi LMB system, which include high operating temperature, high self‐discharge current, low coulombic efficiency, and rapid capacity decay, We developed an electrolyte optimization framework that integrates data collection, machine learning model development, performance prediction, and experimental validation. This ultimately enabled the efficient optimization of electrolyte systems.

**FIGURE 1:**
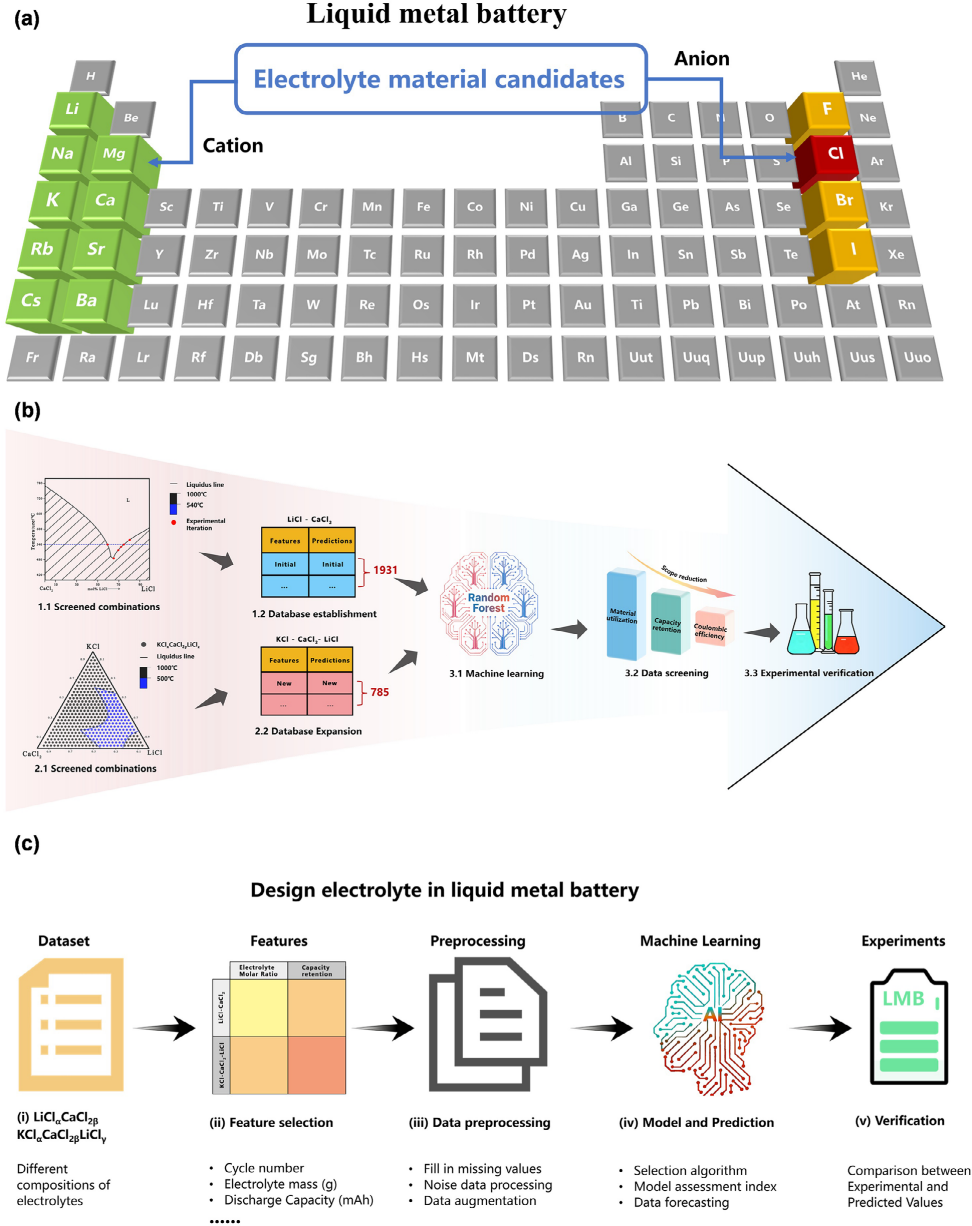
Design of Ca‐based LMB electrolytes: (a) Commonly available cations (green) and anions (yellow and red) selected from the periodic table. (b) Workflow diagram for ML‐assisted electrolyte design: integrating traditional phase diagram design with ML, this approach systematically narrows the proportional range of potential electrolytes. (c) ML flowchart: (i) dataset collection, (ii) feature selection, (iii) data preprocessing, (iv) model training and prediction, (v) experimental verification.

The process begins with the construction of a foundational dataset for the LiCl‐CaCl_2_ binary electrolyte. Owing to the limited availability of experimental data, we first develop a feature‐engineered database that integrates phase diagram information, thermodynamic descriptors, and experimentally measured battery‐level performance. Initial ML‐assisted optimization of the binary composition reveals that binary tuning alone cannot fully address the intrinsic performance bottlenecks of Ca‐based systems. Therefore, we extend the design space to ternary systems by incorporating a third chloride salt. We first select eight common chloride electrolyte salts (Figure 1a). Subsequent multi‐objective screening, based on the criteria of thermodynamic stability, melting point depression, density matching, and cost‐effectiveness, identifies KCl as a promising candidate. We then combined this with phase diagram analysis to select five electrolyte compositions covering both the eutectic region (T_m_<350°C) with CaCl_2_ content less than 10 mol%, and the low‐temperature region with higher CaCl_2_ content (≥25 mol%), assembled the corresponding cells, and performed electrochemical characterization on them. The resulting data (e.g., discharge capacity, coulombic efficiency, cycle life under each composition) are integrated into the original database, providing a significantly expanded training set for subsequent machine learning models. Subsequently, an ML‐assisted electrolyte design workflow is established (Figure 1b). Building upon the binary system research, battery test data from the ternary system (including key parameters such as discharge performance and cycle life under different ternary electrolyte ratios) are incorporated into the initial database to achieve data expansion. The extended dataset enables the ML model to rapidly predict composition domains with optimal performance; subsequently, by combining our screening of desired target features, we experimentally verify representative candidates. Through this iterative loop, we ultimately establish a Ca|KCl‐CaCl_2_‐LiCl|Bi system, exhibiting low operating temperature (480°C), low self‐discharge current (0.31 mA cm^−2^), high coulombic efficiency (>99.5%), slow capacity decay (capacity retention >91% after 100 cycles), and low cost (0.81$ kg^−1^).

The design of the ML workflow (Figure 1c) follows the general paradigm in materials informatics while being tailored to the specific physicochemical correlations in molten‐salt LMBs [20]. The core steps are detailed as follows: (i) Dataset construction: integrating electrolyte physicochemical descriptors (melting point, ionic radius, electronegativity, density, mixing enthalpy, etc.) and experimentally measured LMB performance indicators (coulombic efficiency, discharge energy, capacity retention, material utilization rate); (ii) Feature selection: identifying the most influential electrolyte‐related parameters, creative features, and LMBs’ performance as input variables; (iii) Data preprocessing: including imputation of missing values, data denoising, and data standardization; (iv) Training regression models using the dataset to learn feature‐performance mappings, evaluating model performance through performance metrics, then select an appropriate model to quantitatively predict the performance of candidate electrolyte formulations, and use a progressive screening strategy to narrow down the scope and accelerate the screening process for high‐performance electrolyte formulations; (v) Rapid experimental verification: Conduct a comparative analysis of the predicted values against experimental results to guide subsequent model optimization.

### 2.2 Limitations of the LiCl‐CaCl_2_ Binary Electrolyte System

**FIGURE 2:**
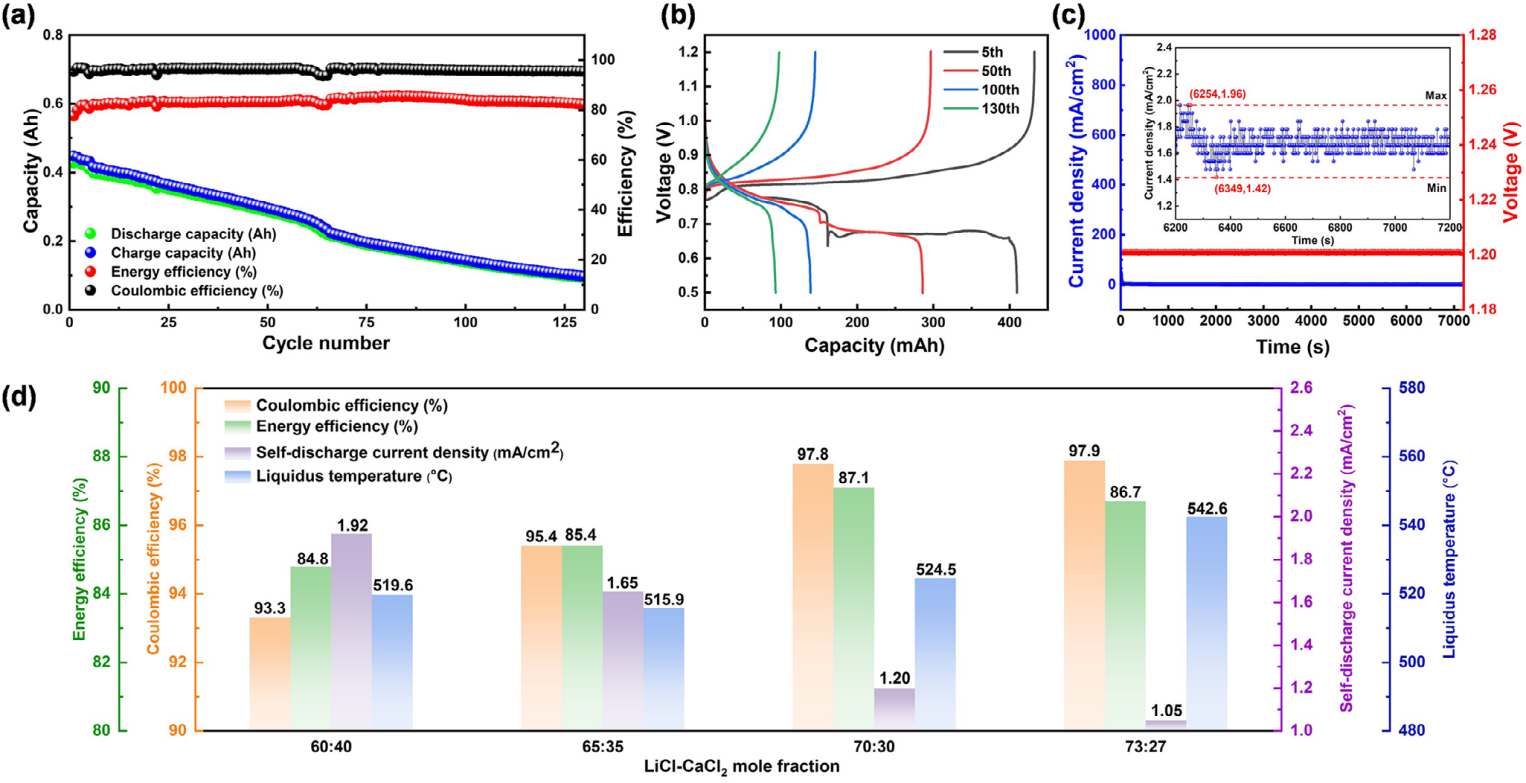
Charge–discharge performance, self‐discharge behavior, and liquidus temperatures of different LiCl‐CaCl_2_ ratios in the Ca|LiCl‐CaCl_2_|Bi battery system. (a) Cycling performance of the Ca|LiCl‐CaCl_2_ (65:35 mol%)|Bi battery, including (b) charge–discharge curves at the fifth, 50th, 100th, and 130th cycles, and (c) self‐discharge current density at the fully charged state. (d) Comparative diagram of key performance metrics (coulombic efficiency, energy efficiency, self‐discharge current density) and corresponding electrolyte liquidus temperatures for Ca||Bi system batteries assembled with different LiCl‐CaCl_2_ molar ratios.

The LiCl‐CaCl_2_ binary molten‐salt system is currently the most widely used electrolyte system in Ca‐based LMBs [14, 16, 17, 18], with the eutectic composition (LiCl‐CaCl_2_ = 65:35 mol%) generally being employed to minimize the melting point. However, the intrinsically high melting temperature and the pronounced dissolution of Ca metal in this melt impose severe limitations, leading to elevated operating temperatures, large self‐discharge currents, diminished coulombic efficiency, and rapid capacity decay. Figure 2a shows the cycling performance of a Ca|LiCl‐CaCl_2_ (65:35 mol%)|Bi cell with a theoretical capacity of 0.5 Ah, operating at 550°C within a 1.2–0.5 V range. The cell delivers a low coulombic efficiency of 95.5%, accompanied by continuous capacity loss, with only 20% of the initial capacity remaining after 100 cycles, which is incompatible with the requirements of grid‐scale storage. The evolution of the charge–discharge profiles (Figure 2b) reveals progressive suppression of the second discharge plateau associated with Ca_11_Bi_10_ formation [15]. By cycle 50, the plateau duration is reduced by half; by cycle 100, it disappears entirely, signaling severe depletion of active Ca.

To ensure data accuracy while maintaining experimental efficiency, the magnitude of self‐discharge was quantified through a 2 h constant‐voltage hold at 1.2 V (Figure 2c). At 550°C, the self‐discharge current density reaches 1.78 mA cm^−2^, equivalent to 1.78% of the applied 100 mA cm^−2^ cycling current. This parasitic reaction correlates directly with the low coulombic efficiency and accelerated capacity fade observed above. To investigate whether compositional tuning within the binary space can mitigate these issues, three non‐eutectic compositions (LiCl‐CaCl_2_ = 60:40, 70:30, 73:27 mol%) were selected based on the reported phase diagram [21] (Figure S1). Their melting points were measured using differential scanning calorimetry (DSC) (Figure S2), and corresponding Ca|LiCl‐CaCl_2_|Bi cells were assembled for self‐discharge tests (Figure S3). As summarized in Figure 2d, increasing the molar proportion of LiCl progressively reduces the self‐discharge current density and improves coulombic efficiency.

To confirm whether the self‐discharge originates from Ca dissolution into the molten salt, x‐ray diffraction (XRD) was performed on electrolytes before and after cycling (Figure S4). The results confirmed that only LiCl and CaCl_2_ phases were present, with no extraneous impurities. Subsequently, the electrolytes from long‐term cycled cells (>480 h) were analyzed using inductively coupled plasma (ICP) (Table S1 and Figure S5). A clear composition‐dependent trend is observed: with initial LiCl fractions of 60, 65, and 70 mol%, the post‐cycling Ca content increases by 3.46%, 2.63%, and 1.91% relative to the initial content, respectively. These results confirm that higher LiCl content effectively suppresses Ca solubility. This behavior is consistent with molten‐salt thermodynamics: elevated Li^+^ concentration lowers Ca^2+^ activity in the melt, thereby reducing the driving force for spontaneous Ca dissolution [15].

Despite these improvements, the binary system remains inadequate. Even at 70:30 and 73:27 mol% LiCl‐CaCl_2_, the self‐discharge current remains above 1 mA cm^−2^, and coulombic efficiency does not exceed 98%. Moreover, deep‐discharge cycling (0.5 V cut‐off) still yields rapid capacity loss (Figure S6). LiCl also increases the electrolyte cost and raises the melting point, hindering efforts toward low‐temperature operation. Therefore, there is an urgent need to expand the design space to multicomponent chloride systems, where additional cations may synergistically regulate Ca activity while enabling significant melting‐point depression.

### 2.3 Determination of the KCl‐CaCl_2_‐LiCl System

In Ca‐based LMBs, molten chlorides are widely used as electrolyte materials owing to their high ionic conductivity and compatibility with liquid electrodes [15]. The design of an electrolyte suited for long‐lived Ca‐based LMBs must satisfy four essential criteria: (1) excellent chemical stability with both cathode and anode materials to suppress side reactions, (2) low melting point to reduce operating temperature and heat management costs, (3) appropriate density to maintain stable liquid‐liquid stratification relative to both electrodes and (4) sufficiently low material cost to enable grid‐scale deployment [5].

Based on these criteria, we first evaluated the chemical stability of Ca with eight candidate chlorides (LiCl, NaCl, KCl, RbCl, CsCl, MgCl_2_, SrCl_2_, BaCl_2_) from a thermodynamic perspective. Previous studies have demonstrated that both MgCl_2_ [22] and NaCl [23] can spontaneously undergo displacement reactions with Ca at elevated temperatures, indicating that they are susceptible to spontaneous reduction by Ca. Such undesirable side reactions consume active Ca metal and degrade battery performance. These two salts were therefore excluded.

Subsequently, the remaining chloride candidates (KCl, RbCl, CsCl, SrCl_2_, BaCl_2_, LiCl) were assessed based on melting point, density, and cost, as shown in Figure S7. BaCl_2_ and SrCl_2_ exhibit extremely high melting points (962°C and 874°C, respectively), far above the target operating temperature window. Their incorporation would not meaningfully reduce the liquidus temperature through eutectic interactions, making them unsuitable for low‐temperature operation. CsCl has a density of 3.99 g cm^−3^, which remains lower than that of the bismuth‐based cathode (approximately 9.78 g cm^−3^). However, during deep discharge cycles, certain discharge products generated at the cathode (e.g., Ca_5_Bi_3_ with a density of approximately 5.3 g cm^−3^ [15]) has close density to CsCl, which may lead to structural instability of the cell. RbCl, while possessing acceptable physical properties, has a prohibitively high unit cost (5274$ kg^−1^), which is also incompatible with the economic constraints of grid‐scale energy storage. After integrating the considerations of thermodynamic stability, melting point, density, and cost, we identified KCl‐CaCl_2_‐LiCl as the optimal electrolyte system for the Ca‐based LMBs.

### 2.4 Machine Learning Design

Artificial intelligence (AI)‐driven materials discovery offers a systematic solution for efficiently exploring chemical space and uncovering nonlinear relationships. This field has developed a comprehensive workflow ranging from multidimensional molecular characterization to high‐throughput virtual screening and directed synthesis, and previous studies have successfully optimized the composition of multicomponent electrolytes using ML, achieving breakthroughs in performance [24, 25, 26, 27, 28, 29, 30, 31]. In this work, by replacing inefficient trial‐and‐error testing with predictive and targeted screening relying on machine learning, electrolyte formulations with superior performance from the KCl‐CaCl_2_‐LiCl ternary system were rapidly identified. A comprehensive feature set (Table 1), incorporating electrolyte‐related parameters, LMB operation descriptors, and creatively designed engineered features, was constructed to support robust model training and performance prediction.

**TABLE 1:** The list of features adopted for machine learning modeling.

Category	Feature	Category	Feature
Creative	Average density^b^	Electrolyte‐related	LiCl mole fraction
	Average density (liquid) [32]		CaCl_2_ mole fraction
	Average surface tension [32]		KCl mole fraction
	Average viscosity [32, 33]		Average melting point [32]
	Average conductivity [32, 34]		Difference in melting point
	Average heat capacity^a^		Average ionization energy^a^
	Average heat capacity(liquid) [32]		Difference in ionization energy
	Average vapor pressure [32]		Mixing entropy
	Average thermal conductivity [32]		Average cryoscopic constant [32]
LMB‐related	LiCl mass		Average ionic radius
	CaCl_2_ mass		Mixing enthalpy(solid)^a^
	KCl mass		Mixing enthalpy(liquid)^a^
	Cut‐off voltage		
	Cycle number		
	Theoretical capacity		

^a^ the electrolyte parameters come from NIST Chemistry WebBook (https://webbook.nist.gov/).

^b^ the electrolyte parameters come from NCBI Pubchem (https://pubchem.ncbi.nlm.nih.gov/compound/).

We first established an initial dataset based on prior studies of the LiCl‐CaCl_2_ molten‐salt binary system. Guided by the LiCl‐CaCl_2_ phase diagram, five compositions with melting points below the target battery operating temperature (550°C) were selected (LiCl:CaCl_2_ = 60:40 mol%; 65:35 mol%; 70:30 mol%; 73:27 mol%; 75:25 mol%). To evaluate battery performance under the non‐molten state, an additional composition for the high‐temperature region with a melting point higher than the operating temperature (LiCl:CaCl_2_ = 81:19 mol%) was further added. We then conducted electrochemical testing on Ca|LiCl‐CaCl_2_|Bi cells with these six electrolyte compositions, measuring electrochemical parameters such as coulombic efficiency, energy efficiency, discharge capacity, and internal resistance under different cut‐off voltage conditions. We treated the electrochemical data from each cycle as a single data point without interval‐based sampling, ultimately obtaining a complete dataset comprising 1931 LMB‐related data entries.

During the collection and evaluation of electrochemical performance data, environmental variations (such as fluctuations in H_2_O/O_2_ levels within the glove box, assembly variability, and variations in the initial activation process of LMBs) could introduce outliers and noisy data into the dataset [20]. To ensure the integrity and reliability of the dataset, these anomalous points were precisely identified and removed through statistical analysis methods during the data preprocessing stage. We took the Coulombic efficiency as the core metric and employed the Z‐score method to screen and remove outliers within the database. For each cell dataset, the coulombic efficiency Z‐score of each cycle was calculated based on the mean and standard deviation of that group's data, and values with |Z| >1.0 were marked as outliers and removed, ultimately eliminating 159 noise data points. The cleaned dataset was then used to train preliminary machine learning models, optimize hyperparameters, and evaluate prediction performance via a grouped cross‐validation strategy based on the composition ratio of battery electrolytes.

To extend the dataset toward the ternary KCl‐CaCl_2_‐LiCl system, we analyzed the ternary phase diagram [32] and selected five representative compositions spanning low‐temperature and eutectic regions (The specific ratio selections are shown in Figure S8). We assembled corresponding cells for electrochemical testing, and obtained an additional 785 points under testing and sampling conditions identical to those of the LiCl‐CaCl_2_ system. Integrating these entries into the original dataset significantly improved the model's predictive adaptability for multicomponent electrolyte systems. Considering the requirements for LMBs in large‐scale energy storage applications (lower operating temperatures, higher coulombic efficiency, lower self‐discharge currents, and slower capacity decay), we selected four key metrics for ML prediction and electrolyte screening: (1) Electrolyte melting point, primarily used to define the composition range during database construction, establishing foundational boundary conditions for subsequent dataset screening; (2) Material utilization M_u_ (calculated as: Mu = C_0_/C_T_, where C_0_ is the initial stable discharge capacity and C_T_ is the theoretical discharge capacity); (3) Coulombic efficiency; (4) Capacity retention rate Cr after 100 cycles (calculated as Cr = C_100_/C_0_, where C_100_ is the discharge capacity after 100 cycles and C_0_ is the initial stable discharge capacity). Among the four metrics above, the validity of the three screening indicators (excluding the electrolyte melting point) was further validated through ML fitting results.

**FIGURE 3:**
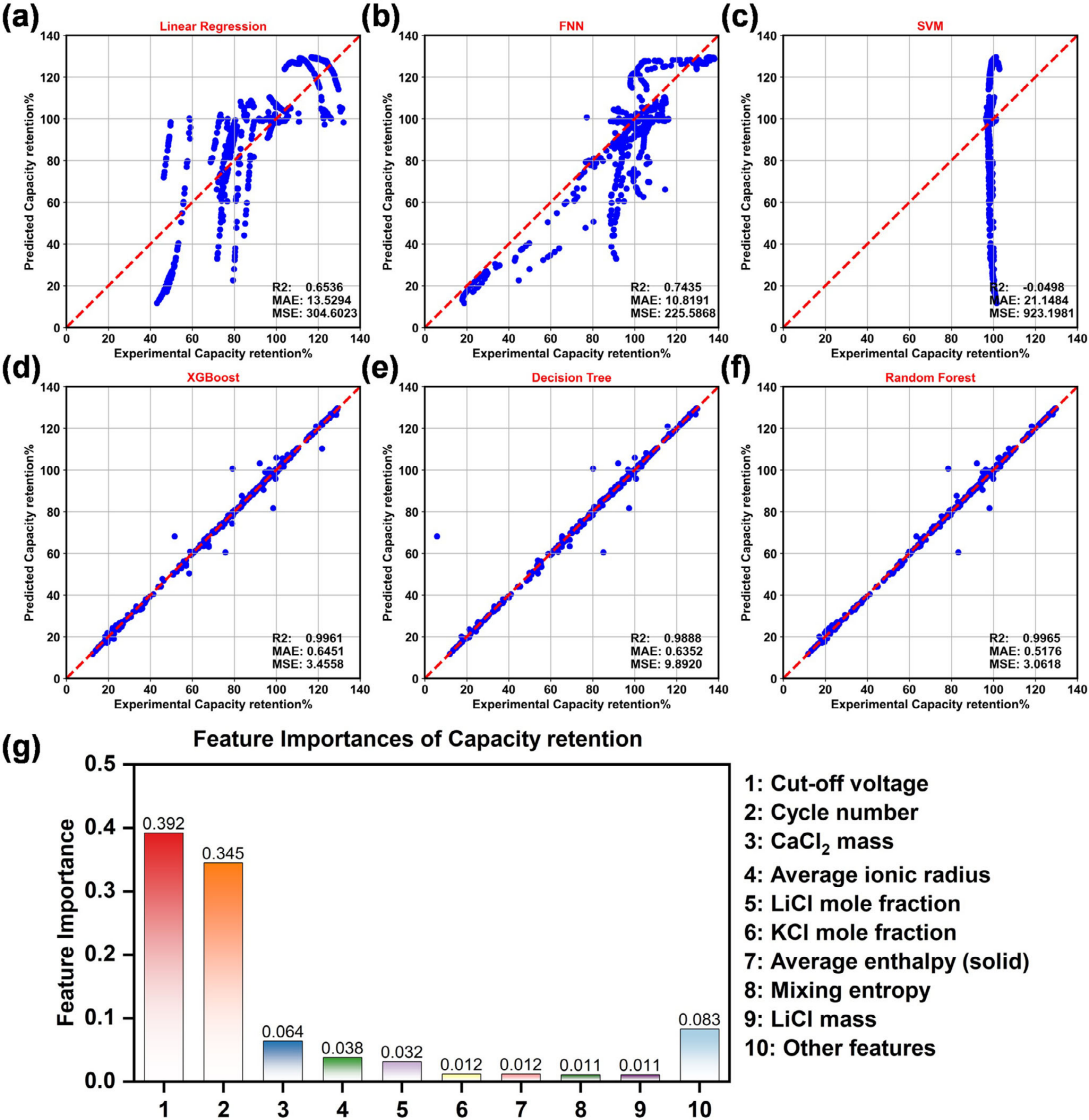
Benchmark results of (a) linear regression, (b) feedforward neural network, (c) support vector machine, (d) XGBoost, (e) decision tree, (f) random forest models, and (g) feature importance analysis in predicting capacity retention of dual‐cation LMBs based on the random forest algorithm.

To identify the optimal predictive model, six ML algorithms were trained using ten‐fold cross‐validation (Details regarding the hyperparameter settings and optimization methods for the relevant models are presented in Table S2). To prevent data leakage caused by autocorrelation between cycles of the same battery, we adopted a grouped cross‐validation strategy. Specifically, we treated the data measured for each electrolyte ratio as an independent group within the same fold, enabling the model to generalize its predictions to unknown electrolyte ratios. The training objectives focused on three metrics: capacity retention rate, material utilization rate, and coulombic efficiency. The training results are shown in Figure 3 and Figures S9 and S10. To avoid potential trade‐offs in multi‐objective output, we trained separate models for each metric to obtain more accurate predictions. The Coefficient of Determination (R^2^) and Mean Absolute Error (MAE) served as primary evaluation metrics, supplemented by Mean Squared Error (MSE) to explain data variations. Figure 3a–c correspond to the fitting results of the linear regression [35], feedforward neural network (FNN) [36], and support vector machine (SVM) model [37], respectively. Linear regression models are commonly used for analyzing data with strong linear correlations. FNN models capture complex data mappings through multi‐layer nonlinear transformations, while SVM models demonstrate high effectiveness in binary classification and regression tasks. In particular, when predicting capacity retention, their R^2^ values were all below 0.9 and their MAE values exceeded 10. This implies that these models exhibit significant prediction errors regarding capacity retention, potentially leading to an electrolyte formulation with an actual capacity retention of only 70% being incorrectly assessed as 80%. Consequently, during subsequent stages of progressive screening, more electrolyte formulations that fail to meet the capacity retention target may be erroneously advanced to the experimental validation phase, thereby increasing validation costs. Therefore, these models are unsuitable for predicting.

Inspired by our previous research [20], we further used XGBoost [38], decision tree [39] and RF models [40] for prediction; the corresponding results are shown in Figure 3d–f. All these algorithms belong to tree‐based ensemble systems and showed markedly improved performance, consistent with the complex, nonlinear nature of LMB electrochemical behavior. Since the Random Forest model demonstrated the highest predictive accuracy in the training results for individual models targeting capacity retention, material utilization, and coulombic efficiency, with R^2^ values exceeding 0.9 and MAE <1, we ultimately selected the Random Forest model as the core predictive model for subsequent electrolyte screening.

Feature importance analysis revealed that cut‐off voltage and cycle number were the dominant factors governing capacity retention, reflecting their roles in phase evolution and long‐term degradation. For material utilization and coulombic efficiency, electrolyte viscosity and ionic conductivity emerged as influential descriptors (Figures S9g and S10g). These insights highlight key physical parameters that warrant future optimization.

After establishing the predictive model, the RF algorithm was first applied in conjunction with the KCl‐CaCl_2_‐LiCl ternary phase diagram to preliminarily screen 1861 low‐melting‐point KCl_(a)_‐CaCl_2(b)_‐LiCl_(c)_ compositions (where a, b, and c represent the molar percentages of KCl, CaCl_2_, and LiCl, respectively, and a+b+c = 100%). To systematically cover the multicomponent combination space, the molar percentages of each component were adjusted in 1% increments, enabling comprehensive exploration of different ratio combinations.

Subsequently, a three‐stage progressive screening strategy was employed to optimize the predicted components: (1) Stage 1: Considering practical application costs and energy density, we set a threshold of >70% material utilization at the 10th cycle and screened 353 formulations with material utilization >70%; (2) Stage 2: Taking a capacity retention rate of over 80% after 100 cycles as the threshold to ensure satisfactory cycling stability of predicted electrolyte formulations, we further screened 74 candidate formulations; (3) Stage 3:To further mitigate the issue of calcium dissolution in the electrolyte, this study adopted a coulombic efficiency >98% as a key evaluation metric. Combined with the results from the previous two screening rounds, 11 optimal electrolyte formulations were ultimately identified.

Some of the 11 shortlisted compositions exhibit only marginal differences in molar ratios (e.g., KCl‐CaCl_2_‐LiCl = 18:35:47 mol% and 17:35:48 mol%), yielding similar thermodynamic and electrochemical behavior. Considering melting characteristics, performance stability, and experimental reproducibility, two compositions (KCl‐CaCl_2_‐LiCl = 25:23:52 mol% and 13:35:52 mol%) were ultimately selected as the most promising electrolyte formulations for experimental verification.

### 2.5 Electrochemical Measurement Verification

**FIGURE 4:**
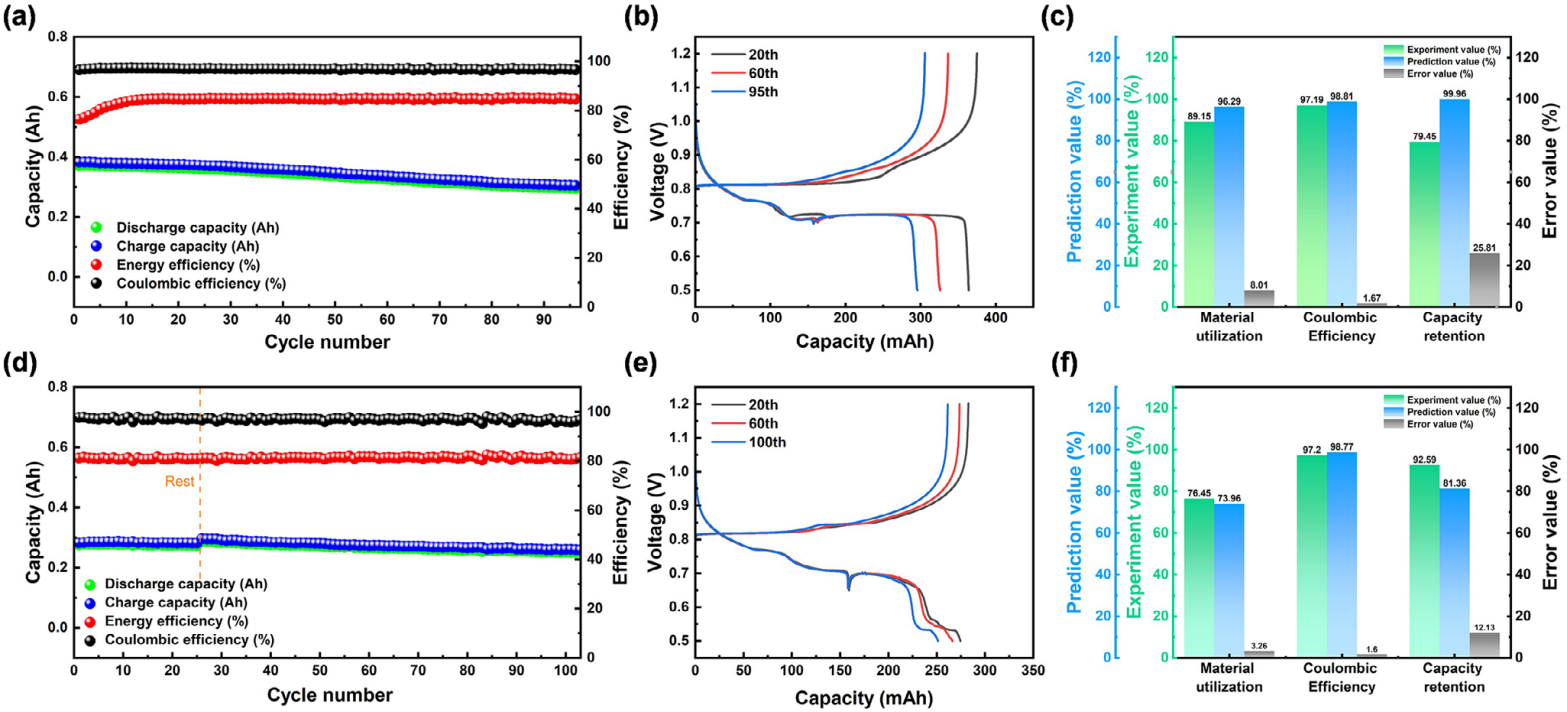
Charge–discharge performance and the comparison between the experiment and prediction in RF of Ca||Bi LMBs assembled with KCl‐CaCl_2_‐LiCl electrolytes at different molar ratios. Cycling performance of (a) KCl‐CaCl_2_‐LiCl = 25:23:52 mol% (d) KCl‐CaCl_2_‐LiCl = 13:35:52 mol% at the current density of 100 mA cm^−2^, with the theoretical capacity of 0.415 and 0.361 Ah. The orange dashed line illustrates the cooling phase, where the operating temperature is cooled to room temperature and maintained for a duration of 24 h. Voltage‐capacity profiles during charge–discharge at different cycle numbers of (b) KCl‐CaCl_2_‐LiCl = 25:23:52 mol% and (e) KCl‐CaCl_2_‐LiCl = 13:35:52 mol% (the cut‐off voltage is 0.5–1.2 V). The operating temperature is 550°C.Material utilization, Coulombic Efficiency and Capacity retention are predicted by RF of (c) KCl‐CaCl_2_‐LiCl = 25:23:52 mol% (f) KCl‐CaCl_2_‐LiCl = 13:35:52 mol%. (The predicted and experimental data for coulombic efficiency and material utilization correspond to the 10th charge–discharge cycle, while the predicted and experimental capacity retention data correspond to the 100th charge–discharge cycle.).

Based on the ML‐guided electrolyte screening results, two Ca‐based LMBs were assembled with Ca as the negative electrode and Bi as the positive electrode. Two ternary molten salts with different KCl‐CaCl_2_‐LiCl ratios were selected as electrolytes, with the following configurations: (1) Electrolyte composition: KCl‐CaCl_2_‐LiCl = 25:23:52 mol%, theoretical battery capacity: 0.415 Ah; (2) Electrolyte composition: KCl‐CaCl_2_‐LiCl = 13:35:52 mol%, theoretical battery capacity: 0.361 Ah. The theoretical capacity was calculated based solely on the Ca anode, assuming Ca_11_Bi_10_ as the terminal discharge phase. Both cells were operated at 550°C to evaluate long‐term cycling stability and charge–discharge behavior under 100 mA cm^−2^ (Figure 4).

The Ca|KCl‐CaCl_2_‐LiCl (25:23:52 mol%)|Bi cell (Figure 4a), delivered a coulombic efficiency >97%, and energy efficiency >85%, with a capacity retention of ∼80% after 100 cycles. Although the capacity fading rate was slightly higher than that of the other ternary system, this composition exhibited a high material utilization (>91%), indicating highly efficient involvement of Ca during cycling. These results demonstrate that this electrolyte composition effectively mitigates self‐discharge and parasitic Ca dissolution compared to the binary LiCl‐CaCl_2_ system. The Ca|KCl‐CaCl_2_‐LiCl (13:35:52 mol%)|Bi cell (Figure 4d) showed superior cycling stability, achieving a coulombic efficiency >97.5% and maintaining >91% capacity retention rate after 100 cycles. Meanwhile, the low Energy efficiency observed in the initial cycles may be attributed to the side reactions, such as those between molten calcium and residual O_2_/N_2_ in the glove box, or those induced by impurities from key components. Notably, its capacity degradation rate was only one‐tenth that of the Ca|LiCl‐CaCl_2_ (65:35 mol%)|Bi cell, highlighting the pronounced enhancement in cycling stability introduced by the ternary electrolyte. During this evaluation, we subjected the fully charged cell to a controlled halt at the 25th cycle, during which both electrodes and the electrolyte underwent complete solidification upon cooling to room temperature. Following a dormancy period of 24 h, the cell was re‐evaluated; no observable degradation in electrochemical performance occurred. This demonstrates remarkable system robustness against thermal cycling and validates the strong structural and chemical stability of the optimized ternary electrolyte under practical operation scenarios.

Additionally, charge–discharge profiles in both ternary electrolyte systems (Figure 4b,e) exhibited two distinct discharge plateaus at approximately 0.77 V and 0.70 V, corresponding to sequential phase transformations during Ca‐Bi alloying. Importantly, unlike the LiCl‐CaCl_2_ binary electrolyte, where the second plateau vanishes rapidly due to accelerated depletion of Ca active material, the ternary electrolytes preserved a clear second plateau even after 100 cycles. This confirms that the optimized ternary compositions effectively suppress Ca dissolution, support stable Ca_11_Bi_10_ formation, and substantially improve long‐term cycling stability.

The Experimental performance of both cells showed strong agreement with ML predictions (Figure 4c,f). The coulombic efficiency prediction error remained within ∼1.6%, while material utilization showed deviations of 3.26%–8.01%, consistent with typical model accuracy for molten‐salt systems. A larger discrepancy was observed for the capacity retention metric. This can be attributed to the fact that the electrolyte primarily influences capacity decay through indirect modulation of Ca dissolution kinetics and interfacial stability, rather than being a direct descriptor of fading behavior. This indirect coupling increases prediction difficulty, explaining the reduced model accuracy for this parameter.

### 2.6 Low‐Temperature Performance Evaluation and Cost Analysis

**FIGURE 5:**
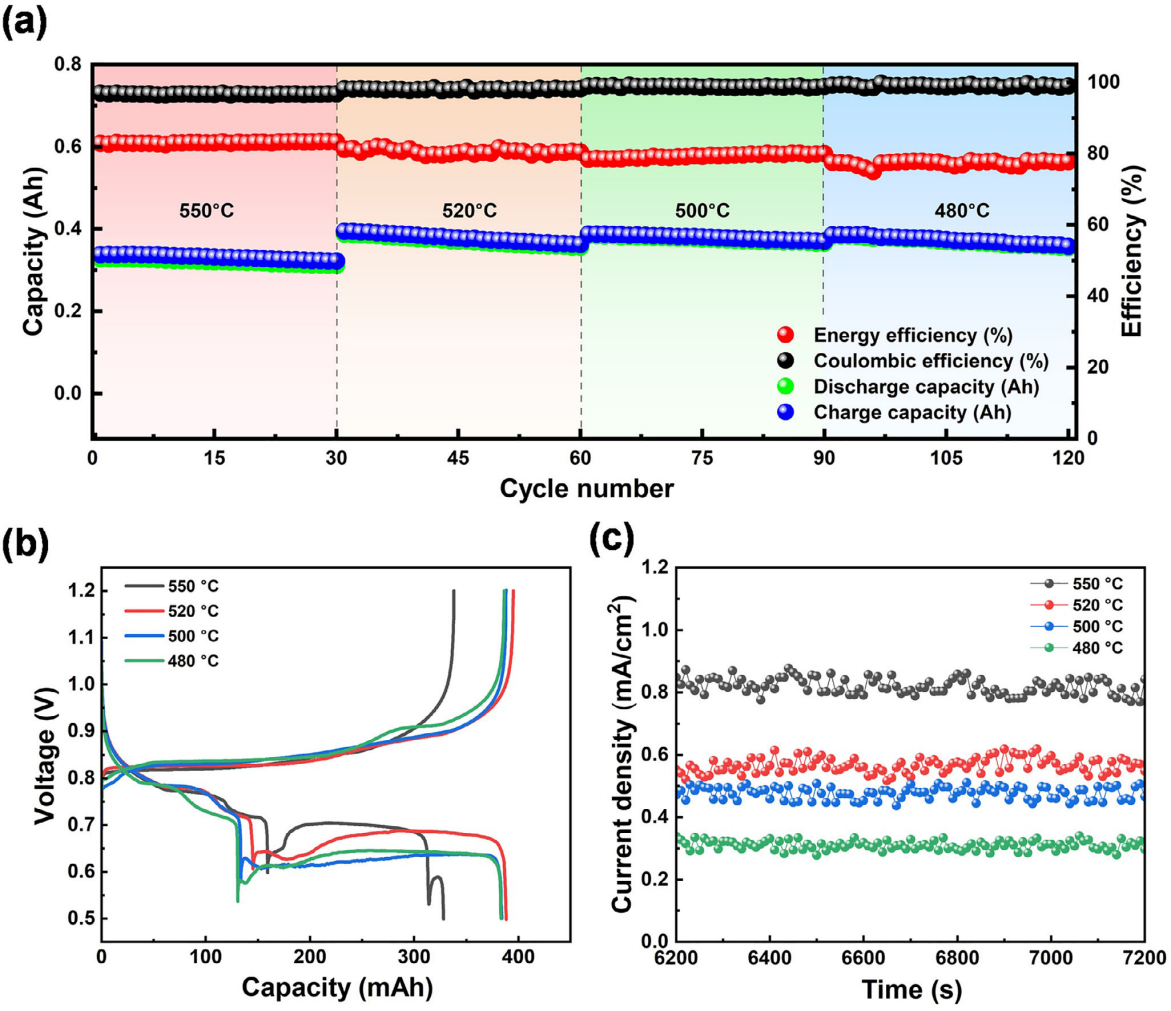
Ca|KCl‐CaCl_2_‐LiCl (13:35:52 mol%)|Bi LMB: (a) Cycling performance test curve at 480°C–550°C and 100 mA cm^−2^ current density. (b) First‐cycle charge–discharge capacity‐voltage curve. (c) Constant‐voltage self‐discharge current density curves at the fully charged state.

According to the DSC results shown in Figure S11, the KCl‐CaCl_2_‐LiCl (13:35:52 mol%) show a liquidus temperature of approximately 461°C, so it is anticipated that the Ca|KCl‐CaCl_2_‐LiCl (13:35:52 mol%)|Bi cell can work at a lower temperature. To systematically assess the influence of temperature on electrochemical behavior, four Ca|KCl‐CaCl_2_‐LiCl (13:35:52 mol%)|Bi cells were subjected to galvanostatic charge–discharge test under 550°C, 520°C, 500°C, and 480°C, respectively, as illustrated in Figure 5. As the operating temperature decreases, the cells exhibited stable cycling accompanied by a gradual enhancement in coulombic efficiency, reaching >99.5% at 480°C. In contrast, a slight reduction in energy efficiency was observed. Analysis of the first‐cycle charge–discharge profiles at each temperature (Figure 5b) revealed progressively increased polarization at lower temperatures. This behavior is likely attributed to suppressed charge‐transfer kinetics and elevated internal resistance under reduced thermal conditions, leading to the observed decline in energy efficiency. Nevertheless, the system still maintained >79% energy efficiency at 480°C, underscoring its robust low‐temperature energy output capability.

To evaluate the self‐discharge characteristics, a 2 h constant‐voltage self‐discharge test was performed on the four cells under fully charged conditions (Figure 5c). The stabilized current values recorded during the final 1000 s were extracted for analysis. At 550°C, the self‐discharge current density was only 0.83 mA cm^−2^, confirming that the ternary‐cation electrolyte effectively suppresses Ca dissolution and aligns with the observed low capacity‐fade rate. Moreover, the self‐discharge current density decreased consistently with lowering temperature, reaching as low as 0.31 mA cm^−2^ at 480°C, equivalent to merely 0.3% of the operational current density (100 mA cm^−2^). This negligible self‐discharge aligns with the trend of increasing coulombic efficiency at reduced temperatures, further validating the enhanced electrochemical stability of the system under lower‐temperature conditions.

In addition to excellent electrochemical performance, the KCl‐CaCl_2_‐LiCl electrolyte demonstrates substantial cost advantages relevant to large‐scale deployment. Based on current raw‐material market prices (Table S3), the electrolyte cost was calculated to be only 0.81$ kg^−‍1^, significantly lower than other reported lithium or calcium‐based molten‐salt electrolytes (Table S4). Overall, the KCl‐CaCl_2_‐LiCl system demonstrates significant improvements over the traditional LiCl‐CaCl_2_ system in terms of both electrochemical performance and cost (detailed comparison is shown in Figure S12). This positions it as a highly promising electrolyte candidate for next‐generation low‐temperature LMBs.

## 3 Conclusion

To overcome the key bottlenecks hindering the practical deployment of Ca‐based LMBs, including elevated operating temperatures, significant self‐discharge, low coulombic efficiency, and rapid capacity decay, this study establishes a research framework integrating data‐driven analysis, ML prediction, and experimental verification. Guided by multidimensional screening criteria encompassing thermodynamic stability, melting point, density, and cost, KCl was identified as an optimal third component, enabling the construction of a ternary KCl‐CaCl_2_‐LiCl electrolyte system. To precisely optimize the electrolyte composition, a comprehensive dataset that couples electrolyte physicochemical properties (e.g., melting point, density) with core electrochemical performance metrics (e.g., coulombic efficiency, capacity retention) was first established. After evaluating multiple algorithms, a RF model was selected due to its superior predictive accuracy, enabling quantitative performance prediction across a broad compositional space. Through a three‐stage progressive screening strategy from material utilization to capacity retention and then to coulombic efficiency, the composition range was gradually optimized, ultimately yielding an optimized electrolyte formulation suitable for large‐scale energy storage applications. Among all ML‐screened and experimentally validated candidates, the resulting Ca|KCl‐CaCl_2_‐LiCl (13:35:52 mol%)|Bi battery exhibits excellent overall performance: stable operation at 480°C, coulombic efficiency exceeding 99.5%, self‐discharge current density as low as 0.31 mA cm^−2^, and a capacity decay rate reduced to one‐tenth that of the initial binary system, while maintaining an extremely low electrolyte cost of 0.81$ kg^−1^. Overall, this work not only delivers a high‐performance, low‐cost electrolyte solution that effectively addresses long‐standing technological challenges in Ca‐based LMBs, but also establishes a new paradigm for ML‐assisted rapid design of energy storage materials. By integrating traditional phase diagram theory with data‐driven algorithms, the proposed strategy dramatically accelerates the discovery and optimization of multicomponent molten‐salt electrolytes, effectively circumventing the inefficiencies inherent to conventional trial‐and‐error approaches. The insights gained here provide both theoretical and experimental guidance for advancing Ca‐based LMB technology and offer a broadly applicable methodology for the rational design of next‐generation molten‐salt electrolytes, thereby contributing to the development of high‐efficiency, large‐scale energy storage systems.

## 4 Experimental Methods

### 4.1 Electrolyte Preparation

All electrolyte salts used in this study are high‐purity materials (LiCl, ≥99%, from Aladdin; CaCl_2_, ≥99%, from Aladdin; KCl, GR, from Sinopharm Chemical Reagent Co., Ltd). The preparation procedure was as follows:(1) All components were weighed according to the target molar ratios inside an Ar‐filled glove box and premixed thoroughly.(2) The mixed salts were dried under vacuum at 120°C and 250°C for 12 h to remove residual moisture. Subsequently, the mixture was heated to 650°C under flowing Ar and held for 7 h to ensure complete melting and homogenization, followed by controlled cooling to 200°C within 8 h. (3) After cooling, the solidified melt was transferred into a glove box and ground into fine powder for subsequent use.

### 4.2 Determination of Electrolyte Melting Point

The melting behavior of LiCl‐CaCl_2_ (60:40, 65:35, 70:30, 73:27 mol%) and KCl‐CaCl_2_‐LiCl (13:35:52 mol%) was characterized using differential scanning calorimetry (DSC, NETZSCH STA 449 F3). Measurements were conducted under an Ar atmosphere at a heating rate of 10 K min^−1^ over the temperature range of 25°C–550°C.

### 4.3 Measurement of Compositional Change of Electrolyte Before and After Cycling

Approximately 2 mg of electrolyte (before and after cycling) was sampled for compositional analysis. Each sample was dissolved in 5 wt.% HNO_3_ inside a centrifuge tube. The concentrations of Ca^2+^ and Li^+^ were quantified using inductively coupled plasma optical emission spectroscopy (ICP‐OES, Agilent 5900), enabling determination of compositional changes during cycling.

### 4.4 Battery Assembly and Testing

Cell assembly (cell structure shown in Figure S13) was performed in an argon‐filled glove box (H_2_O<0.1 ppm, O_2_<0.1 ppm). The positive current collector was a cylindrical stainless steel crucible (SS304) and the negative current collector used iron‐nickel foam (Fe‐Ni = 9:1 mol%). Electrical connections were insulated using ceramic components. Because Ca has a high melting point, batteries were assembled in the fully discharged state. The required quantities of Ca and Bi were weighed, placed into the crucible, heated until molten, alloyed, and then cooled to room temperature. Pre‐dried electrolyte powder was subsequently added. The crucible was then joined with the upper cell housing containing the negative collector, sealed by welding under Ar. After sealing, the assembled cell was transferred to a furnace, heated to 550°C, and held for 12 h to ensure complete Ca‐Bi alloy homogenization.Electrochemical measurements were conducted using a Neware battery tester (CT‐4008‐5V20A‐NFA) within the voltage range of 0.5–1.2 V. Constant current testing evaluated parameters including internal resistance, voltage, capacity, energy, energy efficiency, coulombic efficiency, rate performance, and cycle stability. Constant voltage testing specifically assessed the battery's self‐discharge characteristics.

### 4.5 Machine Learning Section

Machine learning models were implemented in Python using the scikit‐learn package [41]. We employed a suite of algorithms including linear regression (LR), support vector machines (SVM), feedforward neural network (FNN), decision trees (DT), random forests (RF), and gradient‐boosted classifiers (XGBoost). To minimize the influence of sampling order and data variability, ten‐fold cross‐validation (CV) was adopted for performance assessment. Hyperparameters of each algorithm were optimized via grid search to achieve the optimal balance between variance and bias and to avoid both overfitting and underfitting.

The predictive performance of each model was evaluated using the coefficient of determination (R^2^), which quantifies the correlation between predicted and experimental values. R^2^ ranges from −1 to 1, with values closer to 1 indicating a stronger correlation and higher prediction accuracy. R^2^ is defined by:
(1)

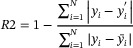

where y_i_ is the actual value, 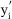 is the predicted value, N is the sample size and ${\bar{y}}_{i}$ is the mean of 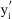, which is defined by:

(2) $${\bar{y}}_{i}=\frac{1}{N}\cdot \sum_{i=1}^{N}y_{i}$$

Mean Absolute Error (MAE) quantifies the average magnitude of absolute differences between predicted and actual values, with a lower MAE indicating greater predictive accuracy. The MAE is defined by:

(3)

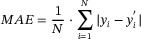

where y_i_ is the actual value, 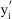 is the predicted value, and N is the sample size.

Mean Squared Error (MSE) is a standard metric used to measure the differences between predicted values and the actual values. It is calculated by averaging the sum of the squares of differences, providing an assessment of the model's fit to the data. The lower the MSE, the closer the model's predictions are to actual values. MSE is calculated by:

(4)

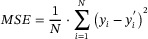

where y_i_ is the actual value, 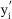 is the predicted value, and N is the sample size.

## Author Contributions

**Xinglin Zhou**: methodology, data curation, investigation, conceptualization, writing – original draft, validation. **Xiaohui Ning**: conceptualization, methodology, validation, supervision, writing – review and editing, project administration, funding acquisition, visualization. **Yan Zhou**: methodology, data curation, investigation, validation, writing – original draft. **Lei Huang**: methodology, data curation, investigation, validation, writing – original draft.

## Conflicts of Interest

The authors declare no conflicts of interest.

## Supporting information




**Supporting File**: advs75994‐sup‐0001‐SuppMat.docx.

## Data Availability

The data that support the findings of this study are available from the corresponding author upon reasonable request.
