# Supplementary material for: Machine Learning‐Assisted KCl‐CaCl2‐LiCl Electrolyte Design for Low‐Temperature, High‐Performance Calcium‐Based Liquid Metal Batteries
Source: Adv Sci (Weinh). 2026 Jun 3:e75994. Online ahead of print. doi: 10.1002/advs.75994 (PMC13336352; doi:10.1002/advs.75994)
Supplement: Supplementary file 1 — Supporting File: advs75994‐sup‐0001‐SuppMat.docx. [file ADVS-9999-e75994-s001.docx]

Supporting Information

**Machine Learning-Assisted KCl-CaCl_2_-LiCl Electrolyte Design for Low-Temperature, High-Performance Calcium-Based Liquid Metal Batteries**

Xinglin Zhou, Lei Huang, Yan Zhou, Xiaohui Ning*

**
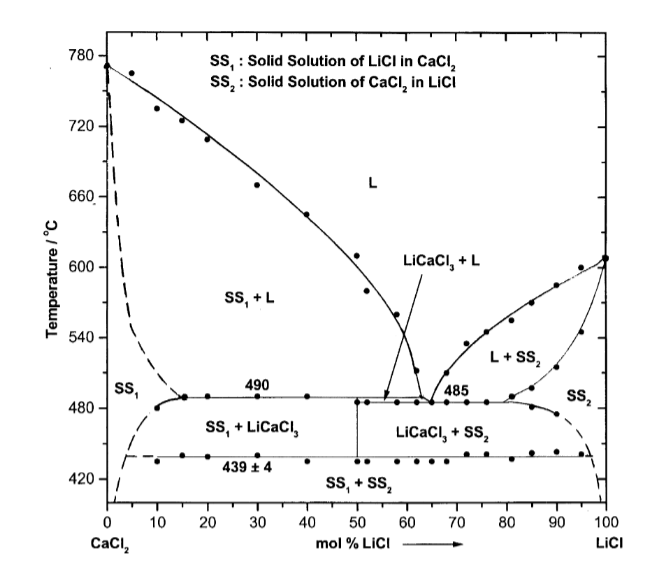
**

**Figure S1.** LiCl-CaCl_2_ phase diagram^[1]^

**
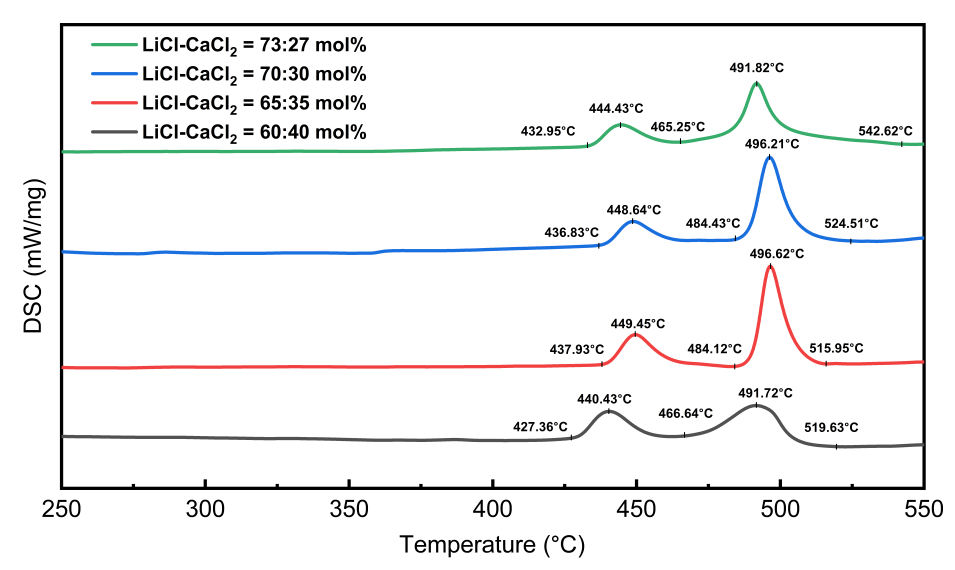
**

**Figure S2.** DSC curves measured for LiCl-CaCl_2_ systems with different molar ratios at 25-550 ℃ with a heating rate of 10 K min^-1^.

**
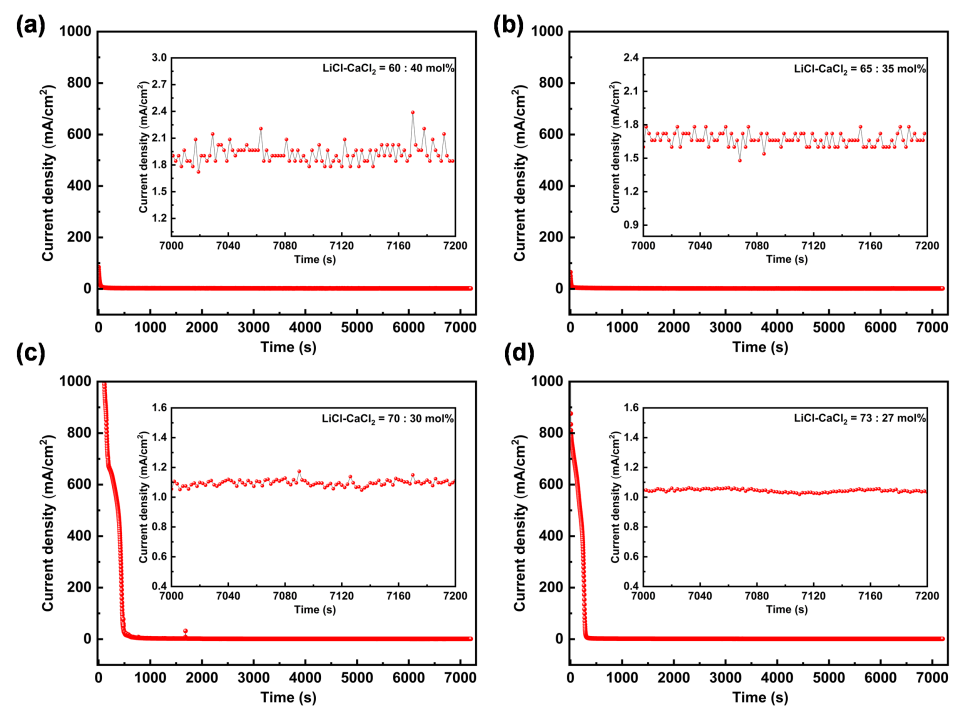
**

**Figure S3.** Self-discharge current density test curves measured after 2 hours of constant-voltage charging at full charge state for Ca||Bi liquid metal batteries assembled with different molar ratios of LiCl-CaCl_2_ electrolyte: (a) LiCl-CaCl_2_ = 60:40 mol%; (b) LiCl-CaCl_2_ = 65:35 mol%; (c) LiCl-CaCl_2_ = 70:30 mol%; (d) LiCl-CaCl_2_ = 73:27 mol%.

**
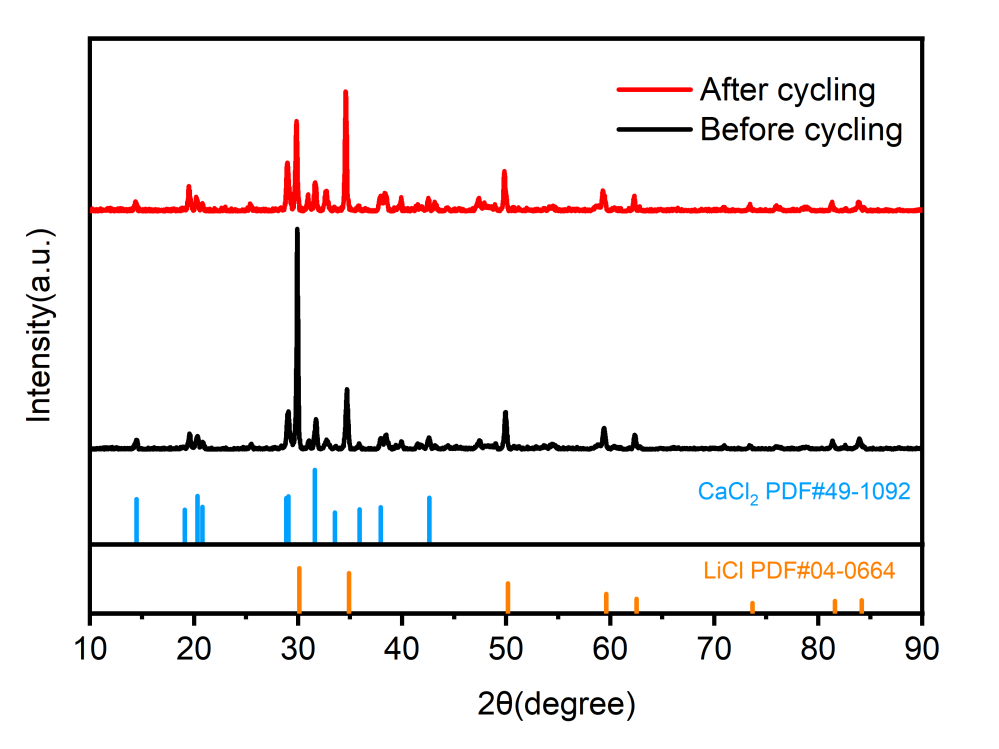
**

**Figure S4.** XRD comparison between the electrolyte after 480 hours of charge-discharge cycling and the pre-cycling electrolyte for the Ca|LiCl-CaCl_2_ (70:30 mol%)|Bi battery.

**
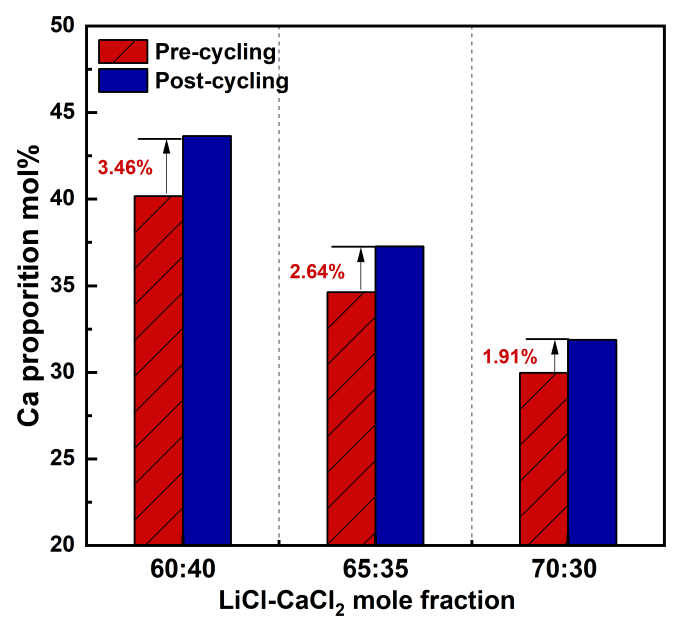
**

**Figure S5.** Variation of Ca proportion in the electrolyte pre- and post-cycling.

**Table S1** The concentration of alkali metal elements in solutions measured by ICP-AES

| Category | Pre-cycling alkali metal concentration  [mg L^-1^] | | Post-cycling alkali metal  concentration  [mg L^-1^] | |
| --- | --- | --- | --- | --- |
|  | Li | Ca | Li | Ca |
| LiCl-CaCl_2_  (60:40 mol%) | 16.73 | 64.88 | 5.82 | 26.02 |
| LiCl-CaCl_2_ (65:35 mol%) | 23.31 | 71.29 | 9.7 | 33.28 |
| LiCl-CaCl_2_ (70:30 mol%) | 28.27 | 69.84 | 26.99 | 72.95 |

**
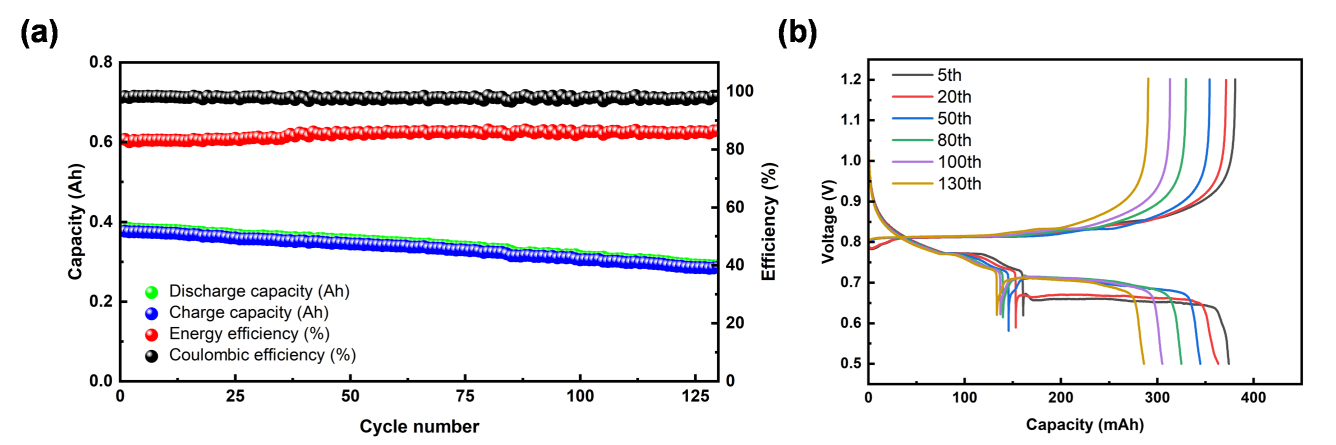
**

**Figure S6.** Charge-discharge performance of Ca|LiCl-CaCl_2_ (70:30 mol%)|Bi LMBs, tested under cut-off voltages of 1.2-0.5 V and a current density of 100 mA cm^-2^: (a) Cycling performance. (b) Voltage-capacity profiles during charge-discharge at different cycle numbers.

**
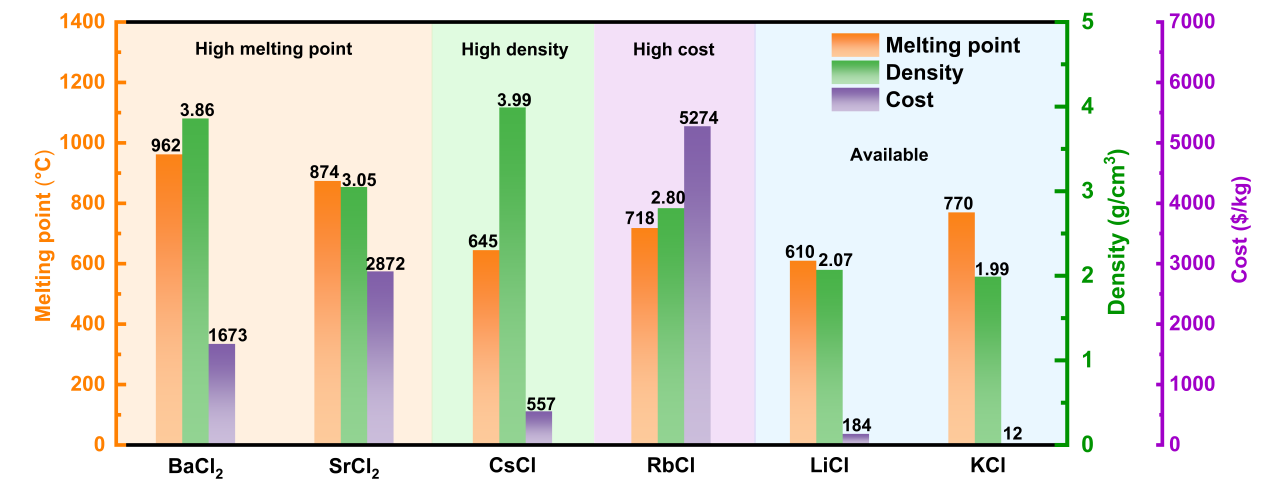
**

**Figure S7.** Properties of different cation chloride salts: melting points, densities, and costs of different chloride salts^[2]^. (cost data sourced from https://www.aladdin-e.com)


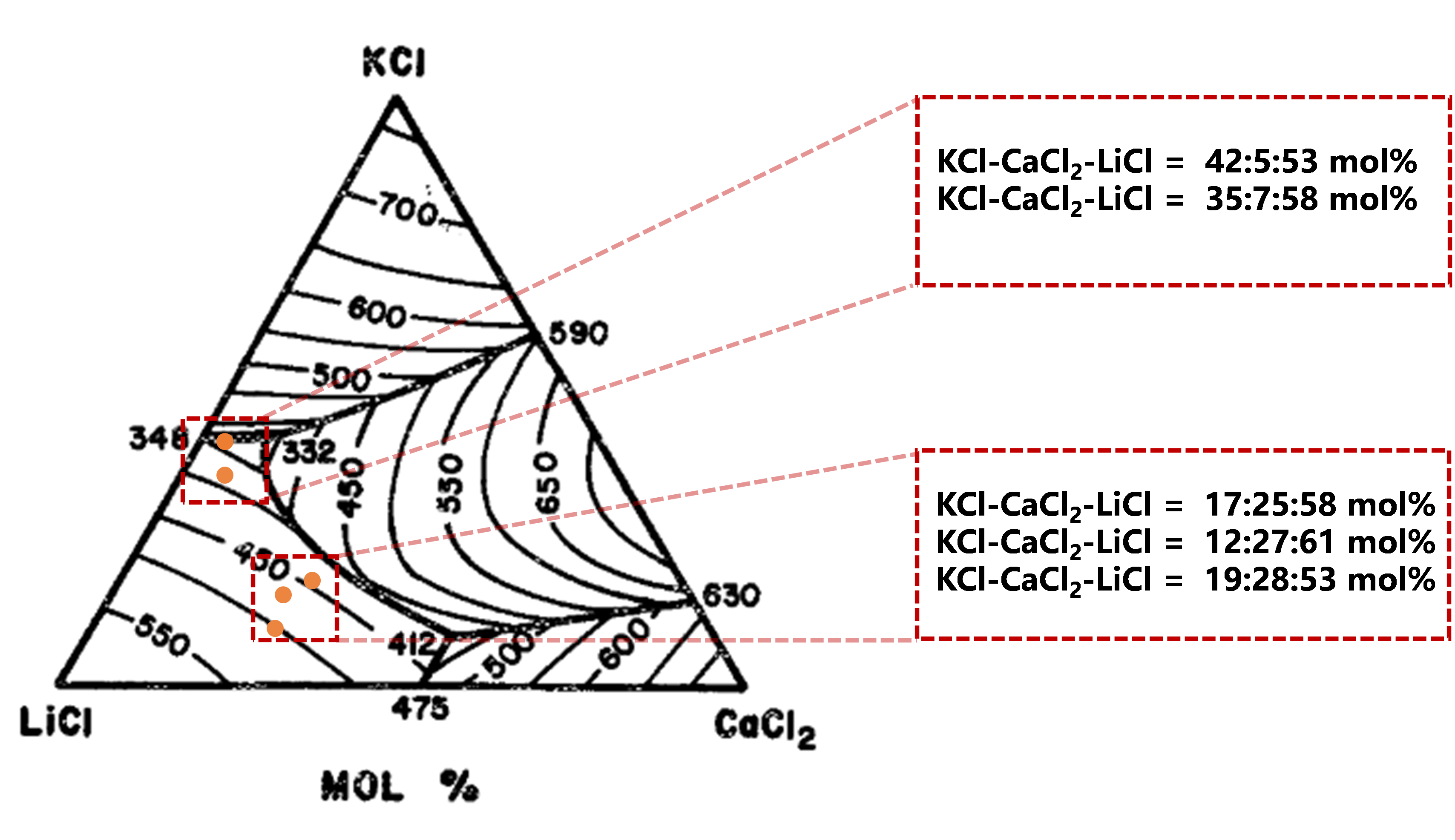


**Figure S8.** KCl-CaCl_2_-LiCl phase diagram^[3]^ and the composition ratios included in the database.

**Table S2.** Hyperparameter settings for different models and optimization methods

| Model | Key Hyperparameters | Values / Range | Optimization Method |
| --- | --- | --- | --- |
| Linear Regression | Regularization Strength (α) | [0.1, 1.0, 10.0, 100.0] | 10-fold cross-validation grid search |
| FNN | Hidden Layer Dimensions | [2n, n, n/2, 1] (n=num_features) | Manual design |
|  | Learning Rate | 0.001 | Default setting of Adam optimizer |
|  | Batch Size | 32 | Empirical selection |
|  | Training Epochs | 100 | Early stopping monitoring |
| SVM | Penalty Parameter (C) | [0.1, 1] | 10-fold cross-validation grid search |
|  | Epsilon Parameter (ε) | [0.1, 0.2, 0.5] |  |
|  | Kernel Function | ['linear', 'rbf', 'sigmoid'] |  |
| XGBoost | Number of Trees | [10, 20, 100] | 10-fold cross-validation grid search |
|  | Learning Rate | [0.01, 0.05, 0.1] |  |
|  | Max Depth | [3, 5, 10, 15] |  |
|  | Minimum Child Weight | [1, 2, 3] |  |
| Decision Tree | Max Depth | [3, 5, 10, 15] | 10-fold cross-validation grid search |
|  | Minimum Samples for Split | [5, 10, 20, 30] |  |
|  | Minimum Samples per Leaf | [1, 2, 4] |  |
| Random Forest | Number of Trees | [10, 20, 100] | 10-fold cross-validation grid search |
|  | Max Depth | [5, 10, 15, 20] |  |
|  | Minimum Samples for Split | [5, 10, 20, 30] |  |
|  | Minimum Samples per Leaf | [1, 2, 4] |  |

**
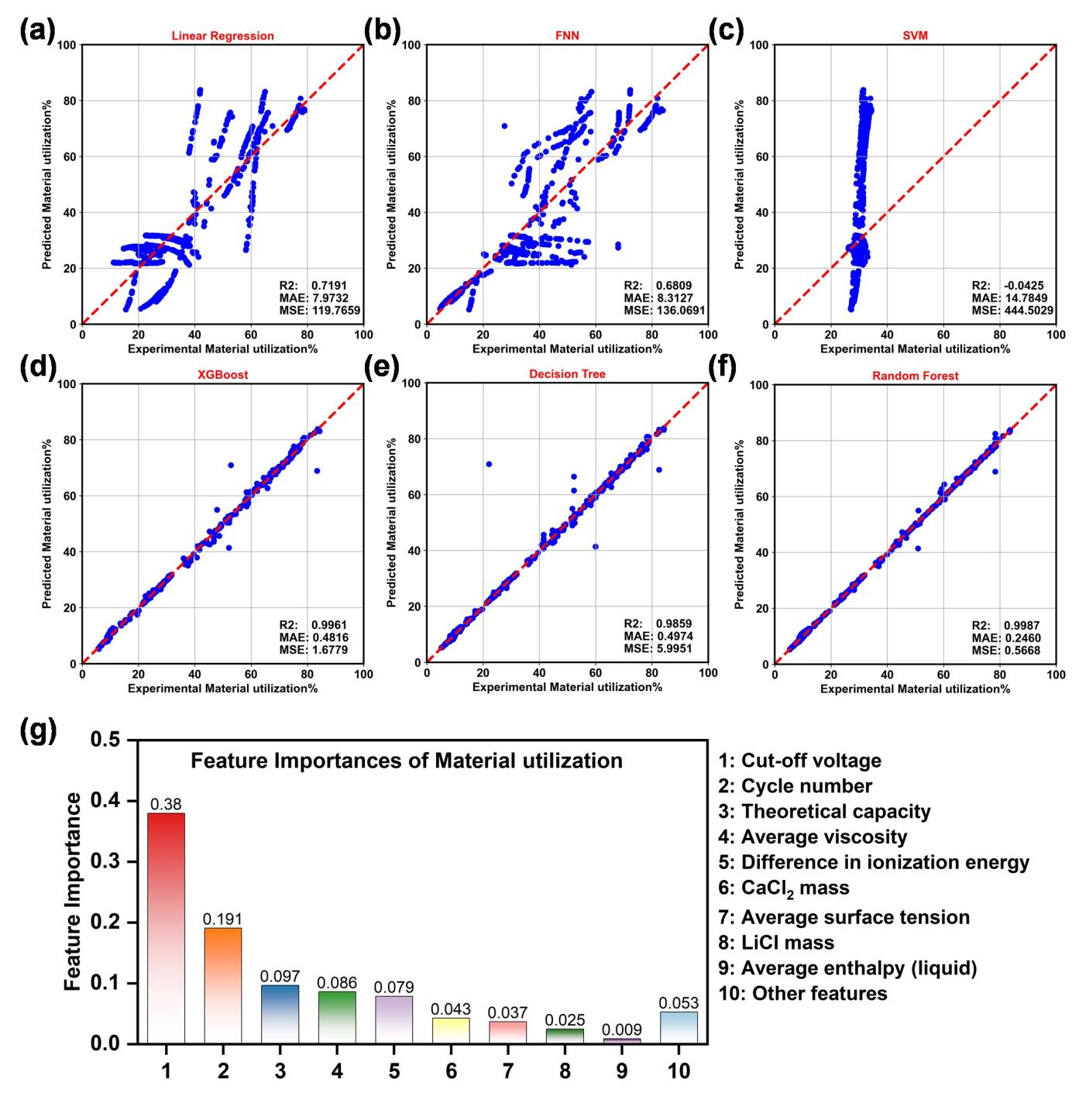
**

**Figure S9.** Benchmark results of (a) linear regression, (b) feedforward neural network, (c) support vector machine, (d) XGBoost, (e) decision tree, (f) random forest models, and (g) feature importance analysis in predicting material utilization of dual-cation LMBs based on random forest algorithm.

**
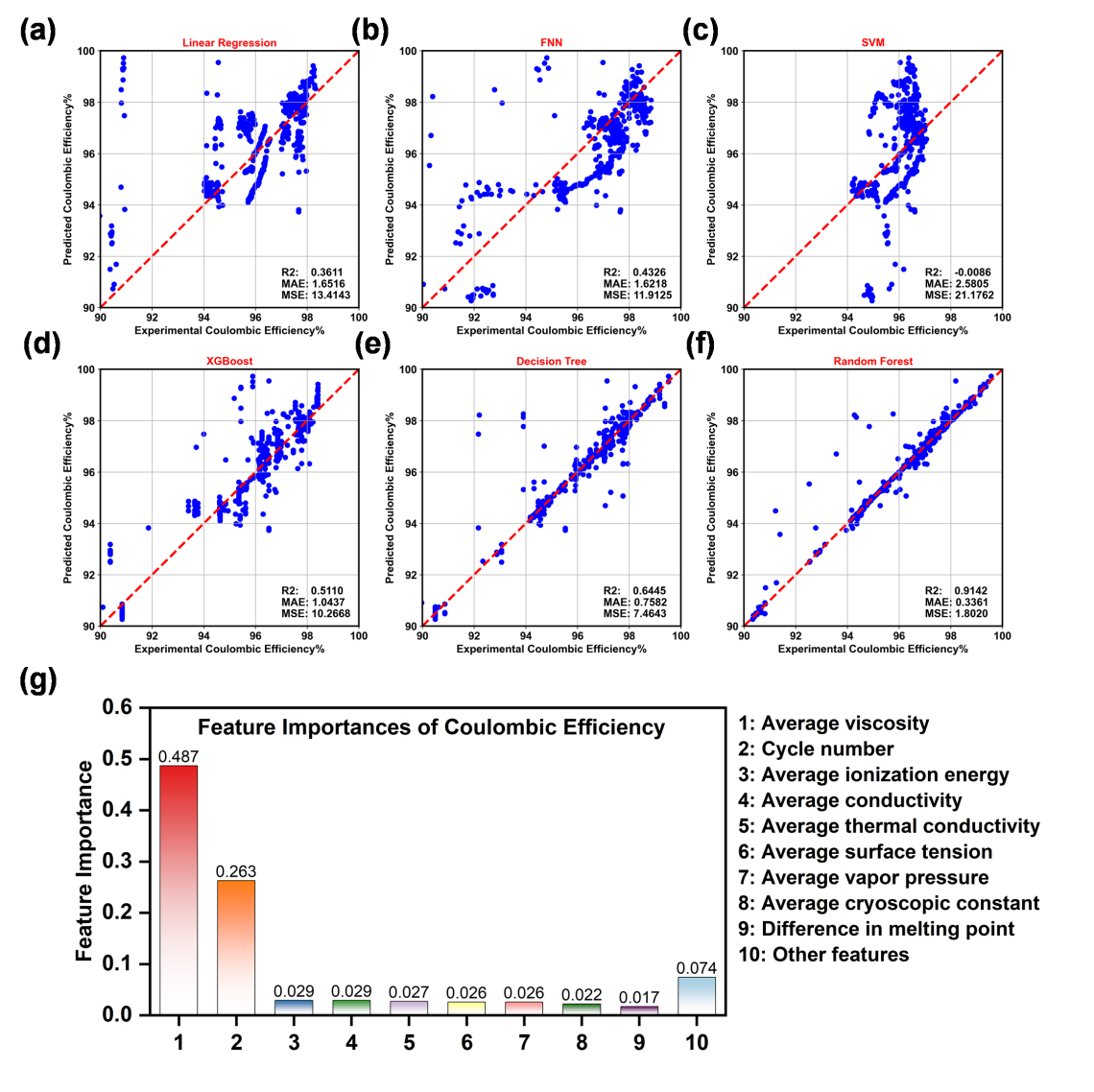
**

**Figure S10.** Benchmark results of (a) linear regression, (b) feedforward neural network, (c) support vector machine, (d) XGBoost, (e) decision tree, (f) random forest models, and (g) feature importance analysis in predicting coulombic efficiency of dual-cation LMBs based on random forest algorithm.

**
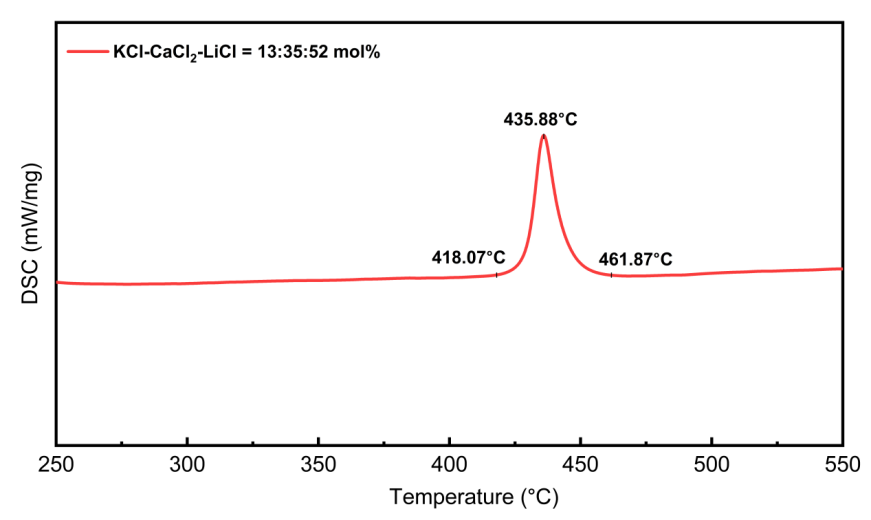
**

**Figure S11.** DSC curves measured for KCl-CaC_2_-LiCl = 13:35:52 mol% measured at 25‒550 ℃ with a heating rate of 10 K·min^-1^.

**Table S3.** Price of typical raw materials used for electrolytes of liquid metal batteries.

| Raw material | Melting point  [℃]^a)^ | Price  [$ kg^-1^]^b)^ |
| --- | --- | --- |
| KCl | 770 | 0.037 |
| LiCl | 606 | 2.000 |
| CaCl_2_ | 782 | 0.340 |
| LiF | 848 | 10.000 |
| LiBr | 551 | 19.800 |
| LiI | 469 | 2.000 |
| KBr | 734 | 1.800 |
| KI | 681 | 10.000 |

^a)^ the electrolyte parameters come from NCBI Pubchem (https://pubchem.ncbi.nlm.nih.gov/compound/)

^b)^ The prices of raw materials are obtained from <https://www.made-in-china.com/.>

**Table S4.** Cost of the designed KCl-CaCl_2_-LiCl electrolyte and some widely used Li-based and Ca-based electrolyte systems^[4]^

| Electrolyte | Component radio  [mol%] | Cost  [$ kg^-1^] |
| --- | --- | --- |
| KCl-CaCl_2_-LiCl | 13:35:52 | 0.81 |
| LiCl-CaCl_2_ | 65:35 | 1.03 |
| LiCl-LiBr-KBr | 33:29:38 | 7.21 |
| LiF-LiCl-LiBr | 22:31:47 | 14.94 |
| LiF-LiCl | 30:70 | 6.70 |
| LiCl-LiI | 36:64 | 2.00 |
| LiF-LiCl-LiI | 20:50:30 | 2.62 |
| iI-KI | 58:42 | 5.79 |


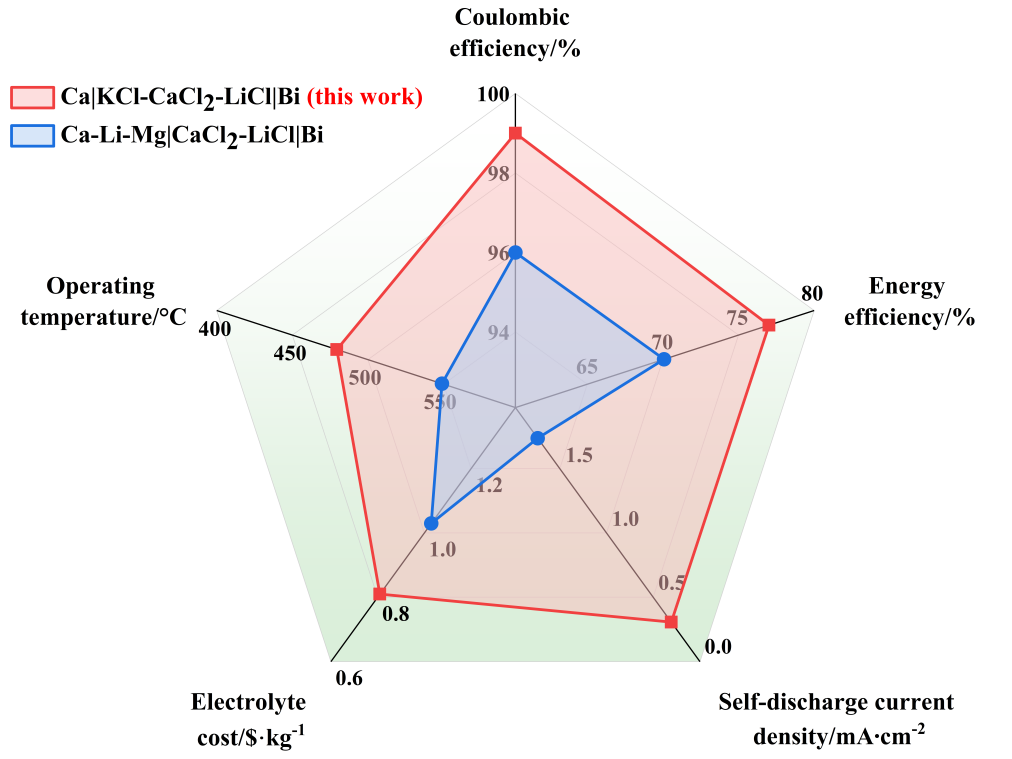


**Figure S12.** Comparison of the performance of the batteries in this study with those reported in the literature for the LiCl-CaCl_2_ system^[5]^

**
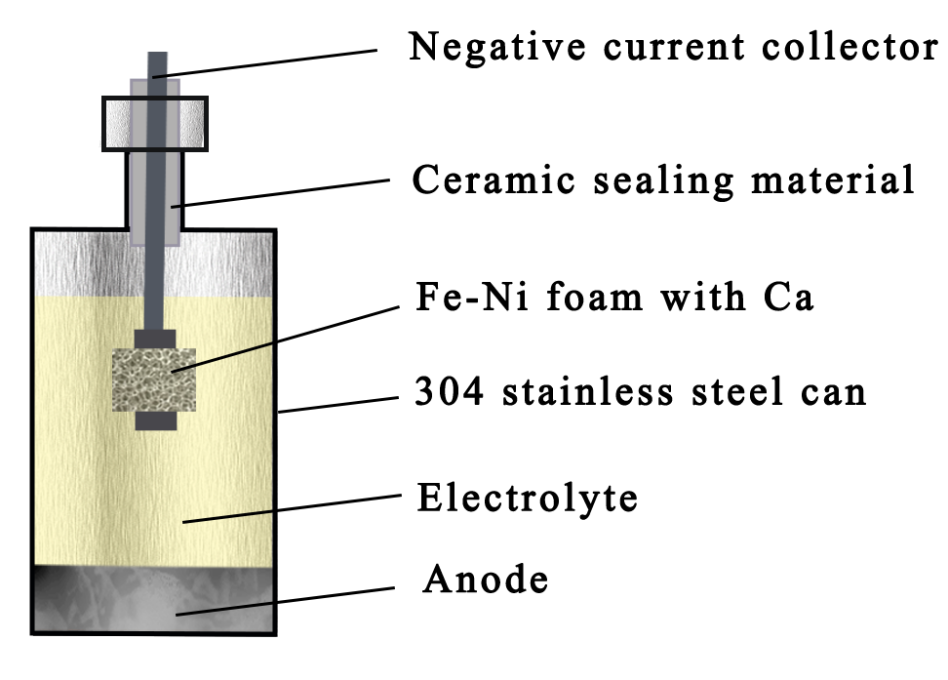
**

**Figure S13.** Schematic diagram of a 0.5 Ah liquid metal battery structure

**References**

[1] K. Mahendran, S. Nagaraj, R. Sridharan, T. Gnanasekaran, Journal of alloys and compounds **2001**, 325 (1-2), 78.

[2] H. Kim, D. A. Boysen, J. M. Newhouse, B. L. Spatocco, B. Chung, P. J. Burke, D. J. Bradwell, K. Jiang, A. A. Tomaszowska, K. Wang, Chemical reviews **2013**, 113 (3), 2075.

[3] G. J. Janz, C. B. Allen, N. Bansal, R. Murphy, R. Tomkins, Physical properties data compilations relevant to energy storage. II. Molten salts: data on single and multi-component salt systems. Rensselaer Polytechnic Inst., Troy, NY (USA). Cogswell Lab.: 1979.

[4] a) T. Ouchi, H. Kim, B. L. Spatocco, D. R. Sadoway, Nature communications **2016**, 7 (1), 10999; b) H. Xie, Z. Chen, P. Chu, J. Wang, Z. Li, H. Zhao, Journal of Power Sources **2022**, 536, 231527; c) K. Wang, K. Jiang, B. Chung, T. Ouchi, P. J. Burke, D. A. Boysen, D. J. Bradwell, H. Kim, U. Muecke, D. R. Sadoway, Nature **2014**, 514 (7522), 348; d) H. Li, K. Wang, S. Cheng, K. Jiang, ACS applied materials & Interfaces

[5] H. Zhou, L. Huang, M. Yu, X. Ning, Energy Storage Materials 2024, 70, 103540.
